# Podoplanin in Inflammation and Cancer

**DOI:** 10.3390/ijms20030707

**Published:** 2019-02-06

**Authors:** Miguel Quintanilla, Lucía Montero-Montero, Jaime Renart, Ester Martín-Villar

**Affiliations:** 1 Instituto de Investigaciones Biomédicas Alberto Sols, Consejo Superior de Investigaciones Científicas (CSIC)—Universidad Autónoma de Madrid (UAM), 28029 Madrid, Spain; luciamontero@iib.uam.es (L.M.-M.); jrenart@iib.uam.es (J.R.); 2 Departamento de Biotecnología-Instituto de Investigaciones Biosanitarias. Facultad de Ciencias Experimentales. Universidad Francisco de Vitoria, 28223 Madrid, Spain

**Keywords:** podoplanin, C-type lectin-like receptor 2 (CLEC-2), ezrin/radixin/moesin (ERM) proteins, platelet, inflammation, thrombosis, lymphangiogenesis, epithelial–mesenchymal transition (EMT), migration, metastasis

## Abstract

Podoplanin is a small cell-surface mucin-like glycoprotein that plays a crucial role in the development of the alveoli, heart, and lymphatic vascular system. Emerging evidence indicates that it is also involved in the control of mammary stem-cell activity and biogenesis of platelets in the bone marrow, and exerts an important function in the immune response. Podoplanin expression is upregulated in different cell types, including fibroblasts, macrophages, T helper cells, and epithelial cells, during inflammation and cancer, where it plays important roles. Podoplanin is implicated in chronic inflammatory diseases, such as psoriasis, multiple sclerosis, and rheumatoid arthritis, promotes inflammation-driven and cancer-associated thrombosis, and stimulates cancer cell invasion and metastasis through a variety of strategies. To accomplish its biological functions, podoplanin must interact with other proteins located in the same cell or in neighbor cells. The binding of podoplanin to its ligands leads to modulation of signaling pathways that regulate proliferation, contractility, migration, epithelial–mesenchymal transition, and remodeling of the extracellular matrix. In this review, we describe the diverse roles of podoplanin in inflammation and cancer, depict the protein ligands of podoplanin identified so far, and discuss the mechanistic basis for the involvement of podoplanin in all these processes.

## 1. Introduction

Inflammation is an inherent protective response that is evolutionary conserved in all multicellular organisms. As a crucial function of the innate immune system, it clears infectious agents and damaged cells, and repairs damaged tissue [1]. Acute inflammation is a self-limiting, transient response that facilitates tissue repair and is beneficial for the organism. However, incomplete, unresolved chronic inflammation could lead to the development of different pathologies, including degenerative diseases associated with aging, fibrosis, and cancer [2,3]. Inflammation involves the activation and chemotactic migration of leukocytes (neutrophils, monocytes, and eosinophils) and mast cells to the site of damage. These cells secrete growth factors, cytokines, and other inflammatory mediators, i.e., histamine, heparin, metalloproteases (MMPs), and serine proteases, which profoundly affect endothelial, epithelial, and mesenchymal cells, stimulating proliferation, differentiation, and migration.

In acute inflammation (wound healing), platelet aggregation and activation occur immediately after tissue damage, and they contribute to accelerating coagulation by forming a platelet plug followed by a fibrin matrix to prevent bleeding and infection by pathogenic microorganisms. The fibrin clot also acts as a reservoir of growth factors released by platelets, such as platelet-derived growth factor (PDGF) and transforming growth factor-β (TGF-β), which are instrumental in attracting neutrophils, monocytes, fibroblasts, and myofibroblasts. These cells, together with the formation of a new extracellular matrix and the induction of neoangiogenesis, facilitate the appearance of granulation tissue. Monocytes differentiate into macrophages in the tissue and, once activated, macrophages represent the main source of growth factors and cytokines that modulate tissue repair. The final phase of healing is re-epithelialization of the wound by proliferation and migration of epithelial cells at the wound edge, a process that requires the dissolution of the fibrin clot and degradation of the underlying collagen by serine proteases and MMPs. Persistence of the causal factors or a failure in resolving the inflammatory response could lead to chronic inflammation, and a large number of clinical and experimental studies linked inflammation and cancer. As a matter of fact, many malignancies arise in sites of persistent infection and inflammation [2,4].

In addition to angiogenesis, the growth of new lymphatic vessels, i.e., lymphangiogenesis, is associated with inflammation and cancer. The main function of the lymphatic vasculature is to drain fluid and macromolecules that leak out of blood capillaries to the interstitial tissue and return back to the blood circulation. It also transports fatty acids and fat from the digestive system. In addition, the lymphatic vascular system plays a crucial role in the immune defense against infection by transporting immune cells from peripheral tissues to the lymph nodes [5]. Lymphangiogenesis is closely associated with wound healing and chronic inflammatory conditions, including psoriasis, rheumatoid arthritis, Crohn’s disease, and ulcerative colitis, and contributes to cancer metastasis [5,6,7]. The lymphatic system helps resolve tissue edema and leads to a rapid activation of adaptive immunity during inflammation. Lymphangiogenesis in primary tumors, on the other hand, facilitates tumor dissemination to regional lymph nodes. Tumor cells can also induce lymphangiogenesis within lymph nodes, creating a lympho-vascular niche that may facilitate the survival of metastatic cancer cells [7]. The cellular events involving lymphangiogenesis are similar to those of angiogenesis and involve stimulation of proliferation and migration of lymphatic endothelial cells (LECs) by growth factors, such as vascular endothelial growth factor (VEGF)-C and VEGF-D that activate a common receptor VEGFR-3. LECs express a number of chemokines that facilitate the transit of immune cells. An example is C–C motif chemokine ligand 21 (CCL21). which remains mostly associated to the cell surface and can bind its receptor C–C chemokine receptor 7 (CCR7) on dendritic cells (DCs). CCR7 is also expressed by tumor cells, and the CCL21–CCR7 axis appears to mediate lymph node metastasis in different types of cancer [7].

Podoplanin (PDPN), also known as PA2.26, gp38, T1α, D2-40, and Aggrus, is a small transmembrane mucin-like glycoprotein whose amino-acid sequence is well conserved across vertebrates. The evolutionary history of its coding gene shows that it evolved in jawed vertebrates about 500 million years ago, and is absent from jawless fishes and non-vertebrate chordates [8]. Podoplanin is widely expressed in different tissues and cell types, such as glomerular podocytes (hence its name), type I alveolar cells, osteocytes, mesothelial cells, choroid plexus, glia cells, some type of neurons, LECs, and different types of fibroblasts. Mice deficient for podoplanin exhibit striking developmental defects, suggesting an important role of this glycoprotein in embryonic development [9,10,11,12]. In adult tissues, podoplanin plays crucial functions in lymphangiogenesis, platelet production in the bone marrow, and the immune response [9,10,11,12], but its precise function in many tissues, such as alveoli, choroid plexuses, mesothelia, and bones remains to be elucidated. Podoplanin expression is upregulated in both epithelial and mesenchymal cell compartments during inflammation and cancer, and a growing body of evidence indicates that it plays an important role in these pathologies.

## 2. Podoplanin Structure and Protein Partners

**Figure 1:**
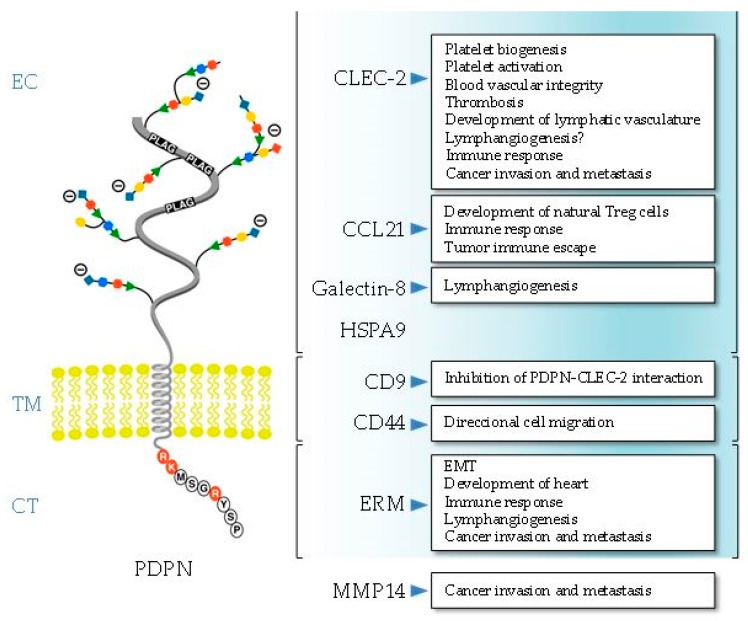
Schematic representation of the structure of human podoplanin (PDPN) showing the amino-acid sequence of the short cytosolic (CT) domain. The ligands and the biological processes in which the interaction with PDPN is involved are presented. The main PDPN structural domain involved in ligand binding is indicated, except for matrix metalloproteinase 14 (MMP14), which is presently unknown. EC, ectodomain; TM, transmembrane region; CT, cytosolic domain.

Podoplanin exhibits the typical structure of a type I transmembrane mucin-like glycoprotein, with a heavily *O*-glycosylated ectodomain, a hydrophobic membrane spanning domain, and a short cytoplasmic tail of only nine amino acids. Since the structure of podoplanin lacks obvious enzymatic motifs, it must exert its cellular functions through protein–protein interactions (Figure 1).

### 2.1. Ectodomain

The sugar modifications at serine and threonine residues of the podoplanin ectodomain are typical of all mucin *O*-glycans and contain galactose linked β3 to *N*-acetyl-galactosamine (GalNAc), called core 1 *O*-glycans, modified by addition of sialic acid [13,14]. The ectodomain holds the critical motifs for podoplanin-mediated platelet aggregation and activation, one of the main biological functions discovered for this glycoprotein so far [11,12,15]. Podoplanin promotes platelet aggregation by interacting with the C-type lectin-like receptor 2 (CLEC-2). CLEC-2 was first identified in platelets as the receptor that promotes aggregation after exposure to the snake toxin rhodocytin [16]. To date, podoplanin is the only known endogenous ligand for CLEC-2. There are three platelet aggregation-stimulating (PLAG) domains repeated in tandem within the ectodomain of podoplanin, and *O*-glycosylation at T-52 in the human PLAG3 domain (as well as *O*-glycosylation at T-34 in mouse PLAG1) appears to be critical for podoplanin-dependent platelet aggregation [17,18]. An additional region in the human podoplanin ectodomain, PLAG4, which is located distant from PLAG1–3, was also recently identified to be involved in CLEC-2 interaction [19]. Cross-linking of CLEC-2 by rhodocytin or podoplanin induces tyrosine phosphorylation of a hemi-ITAM (YXXL) tyrosine-based activation motif of its cytoplasmic tail by Src family kinases. This leads to binding of the tyrosine kinase Syk through its Src homology 2 (SH2) phosphotyrosine binding domains, which initiates a signaling cascade leading to the phosphorylation of linker for activation of T cells (LAT) and SH2 domain-containing leukocyte protein (SLP)-76 adapter proteins and activation of effector enzymes, such as phospholipase Cγ2 (PLCγ2), resulting in platelet aggregation/activation [11,12]. Podoplanin-induced platelet aggregation is involved in the correct separation of the lymphatic and blood vasculatures during development and in cancer metastasis [11,15]. In addition to platelets, CLEC-2 is also expressed in different immune cells, although at low levels, and podoplanin–CLEC-2 interactions play important roles in platelet biogenesis in the bone marrow, as well as in immune surveillance [12].

Another glycosylation-dependent partner interacting with the podoplanin ectodomain is galectin-8, a tandem-repeat type member of the galectin family [20]. It contains two carbohydrate recognizing domains that bind sialyl- and β-galactoside-containing glycans [21]. Both, galectin-8 and podoplanin proteins are highly expressed in LECs [20] and are involved in pathological lymphangiogenesis [22].

Proteomic-based analysis and co-immunoprecipitation experiments revealed that extracellular heat-shock protein A9 (HSPA9) binds the ectodomain of podoplanin on the surface of human oral squamous carcinoma (SCC) cells [23]. Whether or not the interaction between the chaperone and podoplanin is regulated by glycosylation was not investigated. HSPA9 (also known as mortalin) has multiple subcellular locations and is involved in carcinogenesis, stress response, and neurodegeneration [24]. However, the functional relevance of the podoplanin–HSPA9 interaction for any of these processes is unknown.

The extracellular domain of podoplanin is shed from lymphatic endothelial cells into the perivascular stroma, forming a complex with the lymphatic-specific chemokine CCL21, which presumably attracts CCR7-positive immune cells toward the lymphatic vessels [25]. In addition, the podoplanin–CCL21 interaction in fibroblast-like reticular cells (FRCs) present in thymic conduits is associated with the development of specialized T cells [26]. CCL21 is also a potent chemoattractant in the tumor microenvironment, and its binding to podoplanin-expressing cancer-associated fibroblasts (CAFs) is involved in tumor immune escape [27].

### 2.2. Transmembrane Domain

Both the transmembrane domain and cytosolic tail were well conserved during evolution, particularly the N-terminal region containing a GXXXG motif (G^133^IIVG^137^ in human podoplanin) involved in helix–helix oligomerization [8,28]. This motif is also critical for podoplanin association to the detergent-resistant membrane (DRM) fraction or lipid raft microdomains, and was shown to be functionally relevant (see below). Podoplanin is a substrate of presenilin-1 (PS1)/γ-secretase that cleaves its transmembrane domain (between V^150^ and V^151^ in human podoplanin) releasing the intracellular domain into the cytosol [29]. While regulated intramembrane proteolysis of cell surface receptors is an important mechanism involved in different cellular, physiological, and pathological processes [30], the functional relevance of γ-secretase-dependent release of the podoplanin intracellular domain remains to be investigated. Interestingly, both the transmembrane GXXXG motif and the proximal region to the γ-secretase cleavage site were subject of positive selection during evolution [8].

Nakazawa and coworkers [31] reported that podoplanin binds tetraspanin cluster of differentiation 9 (CD9), a cell-surface protein containing four transmembrane domains. The podoplanin–CD9 interaction occurs through transmembrane domains 1 and 2 of the tetraspanin (and involves necessarily that of podoplanin) at specialized membrane regions called tetraspanin-enriched microdomains [32]. The binding of CD9 to tumor cells via podoplanin abolishes its platelet aggregation activity and inhibits podoplanin-induced metastasis [31].

We demonstrated that podoplanin interacts with the standard isoform of the hyaluronan receptor, CD44s, at the surface of SCC cells [33]. CD44 is a highly polymorphic type-I transmembrane glycoprotein that is encoded by a single gene, but gives rise to a large array of variant isoforms (CD44v) in addition to CD44s by alternative splicing [34]. The podoplanin–CD44s interaction is negatively regulated by glycosylation of both protein ectodomains (as only the less glycosylated forms are able to co-precipitate together in immunoprecipitation experiments), and appears to modulate cell migration [33]. Recent data from our laboratory suggest that both the transmembrane and cytoplasmic domains are the structural regions involved in the podoplanin–CD44s interaction.

### 2.3. Cytoplasmic Tail

The cytosolic domain of podoplanin comprises only nine amino acids (RKMS^157^GRYS^161^P for human podoplanin, see Figure 1). The two first juxtamembrane basic residues (RK) are the main ones responsible for podoplanin binding to ezrin and moesin, which anchor the glycoprotein to the actin cytoskeleton [35]. Ezrin and moesin belong to the ERM (ezrin, radixin, moesin) protein family that tethers the actin cytoskeleton to the plasma membrane and is involved in cell polarity, adhesion, and migration [36]. The interaction with ERM proteins is critical for podoplanin-mediated rearrangement of the actin cytoskeleton and modulation of small Rho GTPases, and is involved in promoting epithelial–mesenchymal transitions (EMTs) during embryonic development and cancer [9,10,37]. The podoplanin–ERM interaction is also involved in lymphangiogenesis and the immune response [10], as well as in the recruitment of the glycoprotein by specialized cell-surface protrusions, called invadopodia, implicated in cancer cell invasion [38] (see below).

The cytosolic domain of podoplanin has two serine residues (S^157^ and S^161^ in the human protein) that are potential phosphorylation sites by protein kinases [13,14]. Krishnan and coworkers [39,40] used phosphomimetic and non-phosphorylatable podoplanin mutants to study the functional relevance of podoplanin phosphorylation. They suggest that phosphorylation of S^157^ and S^161^ by the concerted action of protein kinase A (PKA) and cyclin-dependent kinase 5 (CDK5) inhibit podoplanin-induced cell motility. It is tempting to speculate that phosphorylation of these serine residues regulates the podoplanin–ERM interaction, modulating the anchorage of the glycoprotein to the cytoskeleton and activation of Rho GTPases.

## 3. Podoplanin in Embryonic Development and Differentiation

Podoplanin null mice show embryonic lethality due to cardiovascular malformations or die after birth because of a respiratory failure owing to malfunction of alveoli. Heart defects involve impaired myocardial formation because of enhanced E-cadherin expression and downregulation of RhoA GTPase, resulting in abnormal EMT of the coelomic epithelium [41,42]. In addition, podoplanin knockout mice fail to form expanded alveolar sacs at late gestation due to deficient differentiation of type I alveolar cells. These mice show narrower airspaces and die because they are unable to inflate their lungs at birth [43]. While the molecular mechanism behind this phenotype is unknown, altered alveolar differentiation appears to correlate with abnormal expression of some genes, such as ephrinA3 and p21Cip1 [44].

### 3.1. Blood–Lymphatic Vessel Separation

Disruption of lymphatic function was also proposed as a potential cause of the respiratory failure observed in podoplanin null mice [11], as liquid filling the lumen of the developing lung must be cleared out at birth mostly through the lymphatic system. Podoplanin null mice display an abnormal lymphatic vasculature due to defective blood and lymphatic vessel separation [11,12,45]. The mouse lymphatic endothelial cell lineage develops once the blood vascular system is formed at around E9.5, when a subpopulation of venous endothelial cells in the wall of the cardinal vein activates the expression of prospero homeobox 1 (Prox1). Prox1 is a transcription factor that acts as a master regulator of LEC identity. These cells start to express lymphatic endothelial markers, such as lymphatic vessel endothelial hyaluronan receptor (LYVE)-1, VEGFR-3, and podoplanin, and they proliferate and migrate, stimulated by VEGF-C secreted by neighboring mesenchymal cells that activate VEGFR-3/neuropilin-2 signaling to form embryonic lymph sacs from which the entire lymphatic vasculature is eventually derived. Recent data indicate that Prox-1-expressing LEC progenitors, which are not only present in the cardinal vein but also in the intersomitic vessels, leave the cardinal vein via an active process of budding maintaining cell–cell adhesions [46]. At around E11.5, the lymphatic and blood vascular systems become separated by interaction of podoplanin on LECs with CLEC-2 on the surface of platelets. Podoplanin–CLEC-2 binding induces clustering of CLEC-2, activating a downstream signaling pathway involving Src family and Syk tyrosine kinases, the adaptor protein SLP-76, and activation of PLCγ2, as mentioned above [47,48,49,50]. It is worth mentioning that CLEC-2 binds podoplanin with high affinity, and the glycoprotein is able to support platelet capture and activation under arterial rates of shear [51].

*Pdpn*-deficient embryos and neonates show blood-filled lymphatic vessels, edema, and blood–lymphatic misconnections [52,53]. Interestingly, a similar mixed blood–lymphatic vessel phenotype is shared by mice lacking platelet CLEC-2 or Prox1, as well as mice lacking endothelial glycosyltransferase T-synthase, which controls the mucin-type *O*-glycosylation [54,55,56,57]. Podoplanin–CLEC-2-dependent activation of the Syk/SLP-76 signaling pathway in platelets results in the release of granule content and in platelet aggregation that seal the separation zone of lymph sacs and the cardinal vein [53,55]. Platelets inhibit LEC proliferation and migration through the CLEC-2–podoplanin interaction; among the factors released by platelets that appear to play a crucial role in inhibiting LEC function is BMP-9, a member of the bone morphogenetic protein (BMP) subfamily of TGF-β growth factors [58]. There are other potential mechanisms that may be implicated in lymphatic–blood vessel separation mediated by the podoplanin–CLEC-2 interaction, such as prevention of backflow at the lymphovenous junction, and CLEC-2-induced clustering of podoplanin and downstream signaling through ERM proteins [12].

### 3.2. Cerebrovascular Patterning and Integrity

The podoplanin–CLEC-2 interaction is also responsible for the neurovascular phenotype observed in podoplanin null embryos. Podoplanin is expressed in the developing neural tube, and both *Pdpn*- and *Clec-2*-deficient mice show defects on neurovascular integrity in the embryonic brain [59]. These mice show a similar pattern of hemorrhaging since midgestation, which is associated with defective angiogenesis of cerebral blood vessels. Podoplanin on neuroepithelial cells of the developing neural tube binds CLEC-2 on platelets that leak out of surrounding brain vessels, inducing platelet activation and aggregation, thus preventing hemorrhage and facilitating maturation of the developing vasculature by platelet-released factors [59].

### 3.3. Mammary Stem-Cell Function

Podoplanin is expressed in the mammary gland of pubescent or sexually mature virgin and pregnant mice, but is absent from the lactating gland. Podoplanin expression in this organ is restricted to the basal cell layer, including multipotent stem cells [60,61]. A study using a conditional deletion approach through which podoplanin was deleted in embryonic basal cells strongly suggests a role for this glycoprotein in the control of mammary stem-cell function. Loss of podoplanin resulted in a depletion of basal stem cells accompanied by impaired growth and self-renewal potential due to downregulation of Wnt/β-catenin signaling activity [61]. The Wnt/β-catenin pathway plays a major role in controlling the expansion of the basal epithelial cell population after birth [62]. The molecular mechanism behind this phenotype is presently unknown. Likely, it does not involve CLEC-2, as this lectin is absent from the mammary gland, and may implicate interaction with ERM proteins and/or CD44 [61]. This study highlights a role for podoplanin in the biology of mammary stem cells, and establishes this glycoprotein as a novel regulator of Wnt/β-catenin signaling activity. It should be of great interest to ascertain whether podoplanin modulates Wnt/β-catenin signaling in other cell types different from murine mammary basal cells.

### 3.4. Megakaryocyte Growth and Platelet Production

Recently, a novel role for the podoplanin–CLEC-2 interaction was reported in megakaryopoiesis and platelet production in the bone marrow. CLEC-2 is highly expressed in megakaryocytes, the progenitor cell of platelets, and binding to podoplanin-expressing arteriolar FRC-like cells promotes megakaryocyte proliferation and maturation and pro-platelet formation [63]. This process appears to require a reciprocal interaction between megakaryocytes and bone marrow FRC-like cells. The fact that recombinant podoplanin stimulates wild-type but not CLEC-2-deficient megakaryocyte expansion, indicates that a podoplanin-induced signaling pathway downstream of CLEC-2, likely involving activation of the phosphatidylinositol-3-kinase (PI3K)/AKT signaling pathway, is implicated in megakaryocyte proliferation. On the other hand, the CLEC-2–podoplanin interaction stimulates bone marrow FRC-like cells to secrete the chemokine CCL5, which stimulates pro-platelet formation via an unknown signaling pathway [63].

### 3.5. Foot Processes in the Kidney

Podoplanin-expressing podocytes are specialized epithelial cells that cover the basement membrane of glomerular capillary walls. They are so called because of the interdigitating actin-rich foot processes between neighbor cells that anchor podocytes to the glomerular capillary network (also called glomerular tuft). Podocyte foot processes are key determinants of selective permeability through capillary walls and the major size barrier for protein leakage [64]. Thus, podocyte injury is generally associated with proteinuria and often with impaired renal function. Podoplanin is expressed at the apical membrane domain of podocytes facing the luminal urinary side, and loss of its expression is associated with foot process effacement (cell flattening), proteinuria, and decreased glomerular selective permeability in animal models [65,66]. Moreover, podoplanin expression is reduced in angiotensin II-induced injury of human podocytes, a decrease apparently mediated by induction of microRNA (miRNA)-29b [67]. These data support a function for podoplanin in the maintenance of normal podocyte morphology and the establishment of foot processes, which is likely mediated by its anchorage to the actin cytoskeleton through ERM proteins and the control of cytoskeletal organization by regulating the activity of small Rho GTPases.

### 3.6. Development of Natural Regulatory T Cells

Podoplanin null mice also exhibit defects on the maturation of immune cells. Thus, *Pdpn*-deficient mice have a delayed development of natural regulatory T (Treg) cells that result in hyperimmunoglobulinemia E early after birth. Natural Treg cells develop in the thymus as a subset of CD4^+^ T cells, and are devoted to suppressing the immune system in order to maintain self-tolerance and immune homeostasis [68]. A second subset of CD4^+^ T cells, called adaptive Treg cells, develop as a consequence of activation of mature T cells during the adaptive immune response. The phenotype observed in *Pdpn*-deficient mice is apparently due to mislocalizacion of the chemokine CCL21 from the medulla to cortical areas of the thymus [26]. A population of thymic FRCs that form a conduit-like system in the medulla co-express podoplanin and CCL21, and the podoplanin–CCL21 interaction in these cells appears to be critical for the development of natural Treg cells along the cortical–medulla axis during early development and youth of wild-type mice [26].

**Figure 2:**
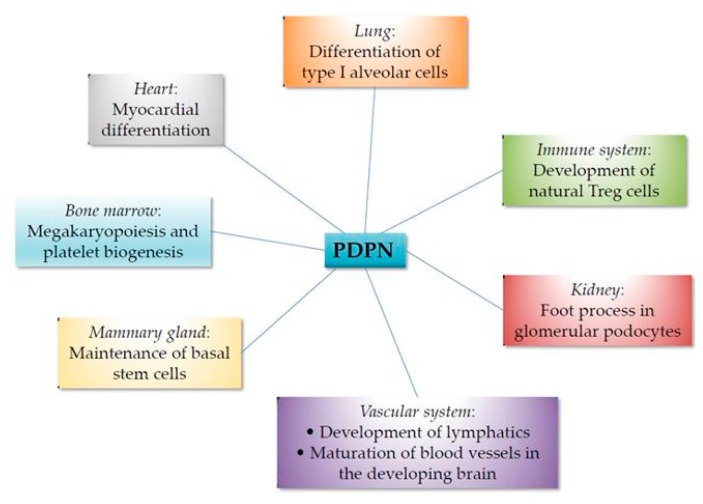
Involvement of podoplanin in embryonic development and differentation.

A summary of the involvement of podoplanin in organogenesis and differentiation is presented in Figure 2.

## 4. Podoplanin and the Immune Response

Antigen-presenting DCs play a key role in initiating immunity against foreign pathogens and maintaining tolerance against self-antigens. They migrate from peripheral tissues to regional lymph nodes via stromal networks, i.e., lymphatic vessels and FRCs, to initiate adaptive immunity and tolerance. To do this, tissue-resident DCs must crawl to and enter blind-ended afferent lymphatic vessels where they move along the lymphatic endothelium to reach lymph nodes. Upon arrival to lymph nodes, DCs penetrate the parenchyma and crawl along FRCs in the paracortex, which is abundant in T cells. FRCs form a dense collagen-based reticular network that supports migratory DCs and T cells and serves as a conduit to transport lymph from the subcapsular sinus to the parenchima [69,70]. Other important non-hematopoietic stromal cells that reside in the lymph nodes are follicular dendritic cells (FDCs). Whereas FRCs control the T-cell zone, FDCs organize the B-cell follicles [71]. Both FRCs and FDCs express podoplanin [72,73]; however, while a pivotal role was reported for this glycoprotein in FRCs, no particular function was ascribed to podoplanin in FDCs.

Podoplanin plays a key function in the intravasation of DCs into afferent lymphatic vessels and DC migration to and within lymph nodes by several mechanisms. Firstly, podoplanin, which is highly expressed in LECs, has the capacity to interact with and immobilize the chemokine CCL21 in the lymphatic endothelium. Then, CCL21 acts as a chemoattractant for migratory DCs, which concomitantly upregulates the chemokine receptor CCR7, promoting DC adhesion to the endothelium and transmigration into the lumen of the vessel [11,74]. Secondly, not only platelets but also leukocytes, including DCs, express CLEC-2, and a deficiency of CLEC-2 in DCs diminish their entry into lymphatic vessels, as well as their migration to and within lymph nodes, implicating CLEC-2 in DC migration. The ligand of CLEC-2 to promote DC migration is podoplanin in both LECs and FRCs, lining frameworks that support migratory DCs from tissues to lymph nodes. The CLEC-2–podoplanin engagement triggers a signaling pathway downstream of CLEC-2 similar to that of platelets that coordinately stimulates Rac1 and reduces RhoA GTPase activity, leading to changes in the dynamics of the actin cytoskeleton that results in DC spreading along stromal cell scaffolds and migration [75]. A question that arose from these observations was whether engagement of CLEC-2 by podoplanin could trigger a signaling pathway downstream of the glycoprotein that could affect FRC or LEC function. As mentioned (Section 3.1), CLEC-2 inhibited LEC migration and proliferation via podoplanin [58]. With respect to FRCs, this issue was confirmed by two reports [76,77]. Under resting conditions, when the probability of FRCs to encounter mature DCs expressing CLEC-2 is scarce, podoplanin induces actomyosin contractility via activation of RhoA/RhoC GTPases, and their downstream Rho-associated protein kinase (ROCK), endowing FRCs with the ability to exert tension within the reticular network. Upon inflammation, engagement of CLEC-2 causes podoplanin clustering in FRCs attenuating podoplanin-mediated contractility, which results in FRC elongation, relaxation of the reticular network, and lymph-node expansion. Lymph-node enlargement represents a critical hallmark of adaptive immunity due to lymphocyte influx and proliferation. Nonetheless, the mechanism via which podoplanin promotes FRC contractility is unclear due to the unexpected finding that the cytoplasmic tail of podoplanin was dispensable for FRC contractility [77].

Kumar and colleagues [78] discovered another face of the DC–stromal axis in activated lymph nodes by showing that DCs maintain podoplanin-positive reticular cell survival to support the ongoing immune response. This effect is mediated by DC-derived ligands of the lymphotoxin β receptor (LTβR), a member of the tumor necrosis factor (TNF) receptor family, which promotes survival of reticular cells by modulating podoplanin expression (Table 1), which, in turn, positively modulates integrin-mediated cell adhesion needed for survival. LTβR induces nuclear factor κB (NF-κB) signaling activity; however, whether or not the NF-κB pathway is implicated in stimulating podoplanin expression was not investigated. The signaling pathway involved in podoplanin-mediated reticular cell survival required activation of RhoA/RhoC–ROCK signaling activity, as well as phosphorylation of focal adhesion kinase (FAK). The fact that this DC–stromal axis occurs only in stimulated lymph nodes during the re-establishment of quiescence and not during homeostasis suggests that reticular cells are in different functional states at homeostasis and in immunized nodes, which could be an important issue to consider in chronic inflammatory diseases [78]. Another interesting point arising from these [77,78] and other [52] studies is that podoplanin, which seems to be excluded from focal adhesions [13], positively regulates integrin-mediated cell adhesion by reorganizing the actin cytoskeleton.

Mice with postnatal deficiency of podoplanin exhibit spontaneous bleeding in mucosal lymph nodes, and bleeding in the draining peripheral lymph nodes after immunization [79]. This is due to decreased vascular integrity of high endothelial venules (HEVs). HEVs are specialized blood vessels that mediate entry of circulating lymphocytes into lymph nodes [71]. Podoplanin expressed in FRCs surrounding HEVs is involved in maintaining HEV integrity by activating platelet CLEC-2 and inducing the release of sphingosine-1-phosphate from platelets, which promotes expression of the junction protein VE-cadherin (the main cell–cell adhesion receptor of endothelial cells) in HEVs, which seal the leakage caused by transmigrating lymphocytes [79]. In addition, podoplanin-expressing FRCs were shown to control B-cell homeostasis in mouse lymph nodes. These cells are the primary source of B-cell activating factor (BAFF), a critical cytokine for peripheral B-cell maturation [80]. In the placenta, BAFF is produced by podoplanin-positive decidual stromal cells stimulated by inflammatory cytokines, such as interferon (IFN)- or IFN-α, and IFN-γ stimulation increases the number of decidual stromal cells expressing podoplanin [81]. Thus, podoplanin may be important for regulating immune responses in the placenta during inflammation; however, whether or not podoplanin is directly involved in BAFF secretion or B-cell homeostasis is unknown.

## 5. Podoplanin in Inflammation-Driven Thrombosis

Deep vein thrombosis and its major complication, pulmonary embolism, are the main cause of cardiovascular death in humans. Venous thrombosis is tightly linked to inflammation, and it is known that platelet depletion protects against deep vein thrombosis in mice [82]. Payne and coworkers [83] demonstrated a critical role of the podoplanin–CLEC-2 axis in thrombosis induced by stenosis in the inferior vena cava. *Clec-2*-deficient mice exhibited a complete protection against thrombosis, while platelet-specific knockout mice showed only a partial protection, suggesting that other cells in addition to platelets mediate the pro-thrombotic effect of CLEC-2—perhaps neutrophils. Podoplanin is upregulated in the wall of the inferior vena cava during thrombosis, localized in the vicinity of the abluminal side of the endothelium, allowing the interaction of platelets with the glycoprotein in the venous wall. The presence of podoplanin in this location contributes to exacerbate deep vein thrombosis, as demonstrated by the fact that treatment with a neutralizing antibody resulted in smaller thrombi [83]. However, the nature of the cell expressing podoplanin in the vessel wall was not investigated.

Podoplanin was identified as a marker of a subset of highly phagocytic F4/80^+^ macrophages in the spleen and peritoneal cavity [84]. These podoplanin-expressing cells seem to represent inflammatory but not resident macrophages, as only the inflammatory cytokine TNF-α or a pro-inflammatory stimulus, such as lipopolysaccharide (LPS), was able to upregulate podoplanin expression in macrophages [85]. A summary of cytokines and other molecules stimulating podoplanin expression in different cell types is presented in Table 1. Furthermore, only podoplanin-positive macrophages were able to bind and activate platelets via CLEC-2, suggesting a role of the podoplanin–CLEC-2 axis for extravascular platelet activation during clotting, i.e., wound healing and inflammatory processes such as atherosclerosis. In this respect, a postmortem immunohistochemical analysis in abdominal aortas localized increased podoplanin expression in smooth muscle cells and macrophages associated with advanced atherosclerotic lesions and necrosis [86]. On the other hand, Hitchcock and colleagues [87] reported that podoplanin is upregulated in subsets of macrophages adjacent to the blood vasculature during inflammation in the liver after systemic infection with *Salmonella typhimurium*. Inflammation triggered by bacteria infection directly induces thrombosis, and both host responses are linked to IFN-γ- mediated enhanced numbers of podoplanin-expressing monocyte cells in the hepatic parenchyma and perivascular sites, resulting in the activation of platelets leaked out from damaged vessels via CLEC-2 [87].

## 6. Podoplanin in Tissue Injury and Fibrosis

### 6.1. Skin

In normal skin, podoplanin is expressed in lymphatic vessels, the basal cell layer of sebaceous glands, and the outer root sheath of anagen hair follicles, but not in the interfollicular epidermis [88,89]. Interestingly, podoplanin expression in hair follicles correlates with that of CD34, a marker of a stem-cell subpopulation located below the bulge area [90], suggesting a potential role for this glycoprotein in hair cycle dynamics. While podoplanin is absent from normal interfollicular epidermis, it is upregulated in basal epidermal keratinocytes and dermal fibroblast-like cells under hyperproliferative conditions, such as wound healing [88,89,91], psoriasis [89,92], or upon a pro-inflammatory stimulus with the phorbol ester 12-*O*-tetradecanoylphorbol 13-acetate (TPA) [88,93]. Upregulation of podoplanin in keratinocytes is triggered by pro-inflammatory cytokines, such as TGF-β1, IFN-γ, interleukin 6 (IL-6), and IL-22 [89] (Table 1). TGF-β1 triggers podoplanin expression via the Smad pathway, whereas IFN-γ, IL-6, and IL-22 do so via signal transducer and activator of transcription (STAT)-1 and STAT-3 [89] (Table 2).

A recent report used an organotypic skin culture with human HaCaT keratinocytes and dermal fibroblasts to show that overexpression of inhibitory protein of κB family kinase α (IKKα) in keratinocytes resulted in disrupted epidermal architecture with increased proliferation, altered differentiation, and foci of keratinocytes invading the dermis. These effects occurred with concomitant induction of podoplanin expression [94]. IKKα is a member of the IKK complex that activates NF-κB signaling, and is involved in epidermal differentiation and in the pathogenesis of skin diseases, such as psoriasis [95]. These data suggest that, in addition to the STAT and Smad signaling pathways, NF-κB signaling controls podoplanin expression in keratinocytes (Table 2).

Although induction of podoplanin expression correlates with hyperproliferation in the epidermis during psoriasis and wound healing [92], it is likely that its function in keratinocytes is related to cell migration rather than proliferation. Maximal podoplanin expression in the epidermis during wound healing occurs at the time of re-epithelialization, and podoplanin-positive keratinocytes exhibit reduced E-cadherin levels. In addition, the knockdown of podoplanin significantly upregulated E-cadherin expression and inhibited the migration of primary normal human keratinocytes [91]. Conversely, ectopic expression of podoplanin in mouse keratinocytes induced cell migration and EMT without affecting proliferation [13,96]. Interestingly, addition of platelets to primary human cultured keratinocytes, or treatment with soluble CLEC-2 reduced keratinocyte motility, which correlated with decreased RhoA GTPase activity and upregulation of E-cadherin [91]. These results suggest that binding of CLEC-2 to podoplanin triggers an inhibitory signal in keratinocytes to impair migration, as occurs in FRCs to attenuate contractility [76,77] and in LECs to inhibit lymphangiogenesis [58], although there are controversial results with respect to the latter (see below). Thus, platelets might be involved in reducing keratinocyte motility by downregulating podoplanin signaling during the initial phases of wound healing [91]. Taken together, the above results suggest a key role for podoplanin during wound repair. However, mutant mice with a keratinocyte-specific deficiency on podoplanin showed normal re-epithelialization after full-thickness cutaneous wounds [97], suggesting that the glycoprotein is dispensable for epidermal repair in vivo. Further studies are necessary to clarify this issue.

### 6.2. Other Tissues

Platelet CLEC-2 was shown to regulate vascular integrity at sites of acute inflammation [11], and a recent report [98] found that the podoplanin–CLEC-2 interaction protects against lung injury in a mouse model of acute respiratory distress syndrome. Platelet CLEC-2 binds podoplanin on alveolar F4/80^+^ macrophages protecting against lung damage during LPS-induced lung inflammation. The authors showed that, following intra-tracheal LPS administration, there was an increase in the presence of platelets bound to neutrophils within alveoli, and studies with *Clec-2*- and *Pdpn*-deficient mice suggest that CLEC-2 limits neutrophil extravasation into the alveolar spaces, and that podoplanin on hematopoietic cells is required to limit neutrophil chemokine expression and attenuate lung dysfunction [98]. In addition, a podoplanin-positive stromal-cell subpopulation representing a component of the progenitor cell response during liver injury was identified during chronic liver inflammation and fibrosis [99]. Also, enhanced podoplanin expression was reported in myofibroblast-like cells of encapsulating peritoneal sclerosis, a rare life-threatening complication of long-term peritoneal dialysis associated with fibrosis and inflammation [100]. Finally, two studies showed upregulation of podoplanin expression during inflammation in the brain. Firstly, podoplanin was upregulated in reactive astrocytes in two mouse models of brain injury (by needle and ischemia). This population of podoplanin-positive reactive astrocytes was also seen surrounding malignant gliomas [101]. On the other hand, podoplanin was shown to be highly expressed in neurons, but not astrocytes of the brain cortex during neuroinflammation associated with neuronal apoptosis induced by intraventricular injection of LPS in rats [102].

## 7. Podoplanin in Inflammatory Lymphangiogenesis

The absence of a lymphatic vasculature is incompatible with life and a lymphatic dysfunction in the adult organism results in chronic lymphedema and attenuated immune response [5]. These observations illustrate the importance of lymphangiogenesis in normal physiology. Whereas de novo formation of lymphatic capillaries is a normal event during embryogenesis, lymphangiogenesis is associated with wound healing, chronic inflammation, transplant rejection, hypertension, diabetes, and lymph node metastasis in adult life [103]. The main signaling pathway regulating lymphangiogenesis during chronic inflammation is the activation of VEGFR-3 by its ligand VEGF-C [104]. The role of the lymphatic vasculature in wounds is to maintain tissue pressure by draining lymph from the interstitial space and to facilitate the delivery of immune cells into the wound. There are two types of LECs with specialized functions in the lymphatic vasculature. Podoplanin is highly expressed in lymphatic capillaries (also called initial lymphatic vessels) that produce CCL21 and attract CCR7-positive dendritic cells and Tregs, whereas pre-collector lymphatic vessels that secrete CCL27 and attract inflammatory CCR10-positive T lymphocytes express low levels of podoplanin [105,106]. The differential expression of the glycoprotein in these two lymphatic endothelial sublineages appears to be negatively regulated by Notch signaling [107] (Table 2). Other studies showed that pro-inflammatory cytokines such as IL-7 and IL-3 stimulate podoplanin expression in endothelial cells with a mixed phenotype and LECs, respectively [108,109]. IL-3 also induces podoplanin expression in blood endothelial cells (BECs), although at a low level [110] (Table 1).

Several reports found that circulating blood or bone-marrow-derived cells of the monocyte–macrophage lineage are progenitors of LECs in lymphatic neovascularization under pathological conditions. Thus, peripheral blood monocytes were stimulated in vitro to induce podoplanin and other lymphatic endothelial markers by mimicking an inflammatory environment with VEGF-C, fibronectin, TNF-α, IL-3, or LPS [111] (Table 1). Also, CD34^+^ human fetal liver-derived cells expressing CD133, podoplanin, and VEGFR-3, as well as podoplanin-positive mouse bone-marrow-derived cells, were found to behave as LEC progenitors and contribute to postnatal lymphatic neovascularization [112,113]. More recently, Cimini and colleagues [114] found that the podoplanin-positive cell population associated with the lymphangiogenic and fibrogenic responses during myocardial wound repair after infarction is widely heterogeneous and displays epitopes of fibrogenic and endothelial commitment, suggesting an alternate ability of podoplanin-positive cardiac cells to generate lymphatic endothelium and pro-fibrotic cells. In a pioneering work, Maruyama and coworkers [115,116,117] reported the incorporation of mouse CD11b^+^ and F4/80^+^ macrophages into lymphatic vessels in the inflamed cornea. These cells, which express podoplanin, LYVE-1. and VEGFR-3 lymphatic markers, were critical for the development and maintenance of inflammation-dependent lymphangiogenesis [115,116]. In fact, a reduction in the number of these podoplanin-positive cells was associated with delayed wound repair in diabetic mice [116]. Interestingly, podoplanin neutralization with a specific monoclonal antibody (mAb) resulted in suppression of lymphangiogenesis and macrophage infiltration during corneal inflammation and ear wound healing, pointing to podoplanin as a novel therapeutic target for lymphangiogenesis and macrophage-related inflammation [118].

Podoplanin knockdown in human LECs inhibits cell polarization, directional migration, and in vitro lymphangiogenesis via a mechanism involving upregulation of Cdc42 and downregulation of RhoA GTPase activity [119,120]. These results suggest that podoplanin expression in LECs is necessary for lymphatic neovascularization, which requires the linkage of the glycoprotein to the cytoskeleton through interaction of the cytoplasmic domain with ERM proteins in order to modulate Rho GTPases. Whether or not the binding of an external ligand to the ectodomain of podoplanin is also critical for lymphangiogenesis is unknown. An indication that it might be the case is the finding that a recombinant fusion protein comprising the extracellular portion of human podoplanin fused to the Fc region of immunoglobulin G1 (IgG1) (PDPN-Fc) interfered with its function in LECs and inhibited lymphangiogenesis in vitro and in vivo [121]. The transgenic overexpression of soluble PDPN-Fc in mouse skin provoked platelet activation via CLEC-2, causing disseminated intravascular coagulation and thrombocytopenia [121]. This event required *O*-glycosylated T34, as mutation of this residue, considered to be essential for the binding of podoplanin to CLEC-2, reduced platelet activation and its side effects [122]. However, the mutant soluble PDPN-Fc retained the ability to inhibit lymphangiogenesis in vitro and in vivo [122], suggesting that the function of podoplanin in LECs is independent of binding to CLEC-2. The above conclusion is contradictory with earlier reports showing that platelets inhibit lymphangiogenesis [58,123]. Furthermore, the suppressive mechanism of lymphangiogenesis by platelets may be responsible for prolonged inflammation in inflammatory bowel disease. Thus, in mouse and rat models of colitis, platelets became activated as inflammation increased and, upon migration into lymphatic vessels, suppressed lymphangiogenesis and exacerbated colitis [124]. This report is interesting as it describes that not only fetal lymphangiogenesis, but pathological lymphangiogenesis can be modulated by platelets. However, whether the suppressive mechanism of lymphangiogenesis by platelets involves the interaction of podoplanin with CLEC-2 was not afforded. In contrast, Hur and coworkers [125] found that interaction of platelets with podoplanin-positive monocytes triggers lymphangiogenesis through activation of the podoplanin–CLEC-2 axis. The binding of CLEC-2 to the glycoprotein activates extracellular signal-regulated kinase (ERK) and AKT signaling pathways that induce transdifferentiation of monocytes to LECs and secretion of lymphangiogenic cytokines. However, these results are mainly based on in vitro experiments, and it is not clear via which mechanism CLEC-2 activates ERK and AKT signaling pathways in monocytes.

The multifunctional lectin galectin-8 is an essential component of the extracellular matrix that is involved in the regulation of angiogenesis and lymphangiogenesis [21]. As mentioned, galectin-8 was found to bind podoplanin and promote adhesion and haptotactic migration of LECs [20]. In a recent study, Chen and colleagues [22] showed that galectin-8 expression is upregulated in inflamed human and mouse corneas, and that this lectin is a key mediator of VEGF-C signaling and a potent lymphangiogenic factor in vivo via a mechanism involving interaction with podoplanin and integrins.

## 8. Podoplanin in Chronic Inflammatory Autoimmune Diseases

In human chronic inflammatory autoimmune diseases, such as multiple sclerosis, arthritis, and colitis, ectopic lymphoid follicle-like structures ranging from simple T- and B-cell clusters to well-organized follicles reminiscent of germinal centers are often observed in the target organ. Features of these lymphoid structures recalling secondary lymphoid organs include compartmentation of T and B cells and presence of lymphatic vessels and HEVs. These lymphoid structures in non-lymphoid tissues are thought to be key determinants of the pathogenesis of autoimmune diseases [126]. Autoimmune diseases are driven by self-reactive T helper cells Th1 and Th17. Th17 cells are heterogeneous and can differentiate into subtypes with different functional properties depending on the environmental stimuli to which they are exposed. Th17 cells can be highly pro-inflammatory causing irreversible tissue damage during chronic inflammatory autoimmune diseases, but they also can be non-pathogenic and protect against bacterial and fungal infection. In fact, under steady-state conditions, Th17 cells are primarily located in barrier organs such as the skin, intestine, lungs, and oral cavity [127]. The expression of podoplanin was found to be upregulated in Th17 cells and other cell types associated with different autoimmune diseases. However, its role in the pathogenicity of many of these diseases is still unclear (Table 3).

### 8.1. Multiple Sclerosis

Multiple sclerosis is a neurodegenerative inflammatory disease characterized by demyelination, axonal damage, and loss of motor and sensory function, in which ectopic lymphoid follicles are developed in the central nervous system [128]. A postmortem study showed increased expression of podoplanin in the inflamed cerebrum of multiple sclerosis patients [129]. Podoplanin was identified as a specific cell-surface marker that distinguishes interleukin 17 (IL-17)-producing mouse Th17 cells from other polarized T helper cells, such as Th1 (producing IFN-γ) and Th2 (producing IL-4, IL-10, and IL-13) [130,131]. Upon adoptive transfer in mice, Th17 cells induce experimental autoimmune encephalitis (EAE), the mouse model of human multiple sclerosis. The formation of ectopic lymphoid structures in EAE was partly dependent on podoplanin as treatment of Th17 cell-recipient mice with an anti-podoplanin polyclonal antibody (Ab) reduced the number of ectopic lymphoid follicles [130]. Nonetheless, whether a reduction in the number of ectopic lymphoid follicles was due to blockade of podoplanin on Th17 cells or on some other podoplanin-expressing cell type was not investigated. In a subsequent article, the same authors used global and T-cell-specific *Pdpn*-deficient mice to show that podoplanin decreases Th17 cell survival in the central nervous system by reducing responsiveness to survival signals elicited by IL-17, promoting tissue tolerance [132]. Chihara and colleagues [133] proposed that podoplanin acts as a negative regulator of T cells. The glycoprotein forms part of a module of co-inhibitory receptors that is shared by non-responsive T cells in several biological contexts, including chronic inflammation and cancer. This co-inhibitory gene program is driven by the cytokine IL-27. A question remaining is via which mechanism podoplanin inhibits T-cell function and promotes ectopic lymphoid follicle formation.

However, the role of podoplanin on the pathogenicity of Th17 cells is far for being clear. It was reported that human pathogenic Th17 cells express less podoplanin than non-pathogenic Th17 cells, which synthesize high levels of the glycoprotein [134]. These non-pathogenic Th17 cells produce anti-inflammatory IL-10 instead of pro-inflammatory IL-17. Under a pro-inflammatory environment, such as high salt concentration, which promotes the differentiation of Th17 cells toward a pathogenic phenotype, Th17 cells exhibited reduced podoplanin expression and increased production of IL-17 [134]. These results are in contrast to what was reported in mice, and suggest that human podoplanin-positive Th17 cells have a protective role against inflammation, rather than promoting it. In fact, podoplanin-positive T cells distinct from IL-17-positive T cells were identified in the skin lymphocytic infiltrates of patients with candidiasis and psoriasis [127]. It should be taken into account that the transcriptomes of the Th17 cell during priming are different between mouse and human [135]. Nylander and colleagues [134] also showed that binding of soluble CLEC-2 to podoplanin ameliorates the Th17 inflammatory response, which is restored by silencing podoplanin expression. CLEC-2, either soluble or expressed on DCs or platelets, was found to induce IL-10-expressing regulatory T cells from unstimulated naive human CD4^+^ T cells [136], emphasizing again a role for the podoplanin–CLEC-2 interaction in the modulation of the inflammatory response.

### 8.2. Rheumatoid Arthritis

Rheumatoid arthritis is a disease characterized by immune-mediated destruction of articular cartilage and bone due to chronic inflammation of the synovium [137]. In both clinical and experimental rheumatoid arthritis, ectopic lymphoid-like structures often develop at sites of chronic inflammation that correlate with the presence of Th17 cells, including a subpopulation of podoplanin-positive Th17 cells [131,138]. In patients with rheumatoid arthritis, synovial IL-27 expression inversely correlates with development of ectopic lymphoid-like structures. Furthermore, IL-27 inhibits the differentiation of podoplanin-positive Th17 cells, and the number of podoplanin-positive Th17 cells is enhanced in mice deficient for the IL-27 receptor, which develop inflammatory arthritis [138]. These results suggest a role for IL-27 as a negative regulator of ectopic lymphoid-like structures in rheumatoid arthritis by controlling effector T cells, as also seen in cancer and other models of chronic inflammation [133].

In addition, increased podoplanin expression was observed in fibroblast-like synoviocytes and macrophages of rheumatoid arthritis patients with respect to patients with osteoarthritis and normal synovial tissue [139,140,141,142]. Podoplanin expression in synoviocytes also correlated with the presence of ectopic lymphoid structures and inflammation [140,143]. In fact, podoplanin-positive FRCs distinct from FDCs were observed associated to ectopic lymphoid follicle-like structures and inflammation in mice and humans in a variety of tissues, such as the pancreas, kidney, liver, and salivary gland [144]. Synovial fibroblasts represent a heterogeneous cell population that becomes activated under persistent inflammation. They secrete pro-inflammatory cytokines and chemokines, invade and destroy articular cartilage, and cause bone erosion by stimulating osteoclasts [145]. Podoplanin-positive human synovial fibroblasts exhibit characteristics of highly invasive myofibroblasts [139,146] and are able to attach to, invade, and degrade cartilage in a severe combined immunodeficiency mouse model of rheumatoid arthritis [141], suggesting that podoplanin-positive synoviocytes are actively involved in joint destruction. Ekwall and colleagues [139] found that podoplanin expression was induced by the pro-inflammatory cytokines IL-1β, TNF-α, and TGF-β1 in cultured primary synovial fibroblasts, and this result was confirmed by Croft and coworkers [141] for IL-1β and TNF-α, but not for TGF-β1 (Table 1).

Secretion of pro-inflammatory IL-17 appears to be regulated by podoplanin-mediated cell–cell interactions between immune cells and synoviocytes, as found in co-culture experiments with phytohemagglutinin-activated peripheral blood mononuclear cells (PBMCs) and synoviocytes isolated from patients with rheumatoid arthritis. IL-17 secretion was dependent on T-cell activation and interaction of T cells with synoviocytes, and was highly reduced by incubation of either PBMCs or synoviocytes with an anti-podoplanin Ab [147]. Nevertheless, as both synoviocytes and Th17 cells express podoplanin, it remains to be established which is the ligand of podoplanin in these co-cultures, and in which of these cell types the ligand is located. A role for podoplanin in mediating interactions between activated immune cells and dermal fibroblasts derived from inflamed skin for secretion of IL-17 was also proposed in psoriasis [148].

### 8.3. Systemic Sclerosis

In systemic sclerosis, a disease characterized by autoimmunity, vasculopathy, and fibrosis, CD34^+^ dermal fibroblasts present in normal skin disappear. The loss of this cell population occurs due to a phenotypic conversion from CD34^+^ to CD34^−^/CD90^+^/podoplanin-positive fibroblasts, which seems to represent a common fibroblast response to inflammation and a shift to a tissue repair program [149]. Primary human dermal cultured fibroblasts were found to upregulate podoplanin expression in response to inflammatory cytokines TNF-α and IL-1β (Table 1). Maximal podoplanin induction was obtained via the combination of TNF-α and an anti-LTβR agonist Ab [149].

### 8.4. Other Autoimmune Diseases

Increased podoplanin immunostaining was found in the salivary glands of patients with primary Sjögren syndrome, an autoimmune disease of the exocrine glands of unknown ethyology. This enhancement was associated with higher numbers of lymphatic capillaries, as well as the presence of podoplanin-positive myoepithelial cells surrounding the ductal epithelium, and lymphoid aggregates in the periductal area that might correspond to FDCs and/or Th17 cells [150]. On the other hand, downregulation of podoplanin messenger RNA (mRNA) expression was found in the kidneys of mice with pristane-induced lupus nephritis, which in humans is one of the most problematic complications of systemic lupus erythematosus. The loss of podoplanin expression correlated with increased proteinuria and podocyte foot process effacement [151], as reported in other models of injured kidney [65,66,67]. In patients with the lupus-related autoimmune skin disease, chronic lip discoid lupus erythematosus, immunohistochemical detection of podoplanin in the oral mucosa is associated with increased risk of malignant progression to lip SCC [152], pointing to podoplanin as a marker of bad prognosis in SCC, as described in the next section.

## 9. Significance of Podoplanin Expression in Cancer

Podoplanin expression is upregulated in a large variety of cancers, including angiosarcomas, chondrosarcomas, osteosarcomas, malignant mesotheliomas, germ-cell tumors, gliomas, glioblastomas, and SCCs [9,10,153,154]. Podoplanin is seen in the tumor cells themselves, as well as in stromal cells, particularly in cancer-associated fibroblasts (CAFs). Podoplanin expression in tumor cells is generally associated with poor prognosis, notably in glioblastomas and SCCs of skin, esophagus, and head and neck [9,10,154]. However, podoplanin expression was reported as a good prognosis factor in uterine cervical [155] and lung SCCs [156,157,158]. In SCCs, cells expressing podoplanin are frequently located at the outer edges of tumor nests [14,155,157,159,160], suggesting that cytokines or growth factors secreted by neighboring stromal cells might induce podoplanin expression in the neoplastic epithelium. This assumption was confirmed by a recent report showing that pro-inflammatory cytokines secreted by CD45^+^ stromal cells induce podoplanin expression at the invasive front of cervical SCCs [161]. Podoplanin is also expressed in the hyperplastic and dysplastic epithelia adjacent to primary tumors [14,159,162], an event associated with local invasion in extramammary Paget’s disease [163] (see Section 10.1).

A few reports detected enhanced expression of soluble podoplanin in biological fluids of cancer patients associated with poor prognosis. In bladder cancer, higher levels of soluble podoplanin in the serum of patients are associated with larger and more aggressive multifocal tumors [164]. Likewise, the levels of soluble podoplanin in plasma were found to increase in patients with a wide variety of cancers compared to normal individuals, as well as in patients with metastasis with respect to patients with non-metastatic tumors [165]. Interestingly, the levels of soluble podoplanin were reduced after treatment of patients with chemotherapy or surgery followed by chemotherapy, pointing to soluble podoplanin as a specific cancer biomarker [165]. An unsolved question is the origin of the soluble protein. Soluble podoplanin can be generated after proteolytic cleavage of the extracellular domain and entry into the blood circulation, or can be secreted by both tumor and stromal cells as a full-length protein attached to extracellular vesicles. Several reports found that membrane-bound podoplanin is susceptible to degradation by different types of proteases, including calpains, presenilin-1/γ-secretase, and metalloproteases [29,166,167]. The stability of the protein at the cell surface appears to be controlled by *O*-glycosylation and sialylation. Thus, *O*-glycan-deficient or desialylated podoplanin expressed in LECs is highly susceptible to degradation by metalloproteases (MMPs) [167]. On the other hand, podoplanin was detected in EpCAM-containing microparticles isolated from malignant pleural effusions [168], as well as in microvesicles and exosomes secreted by tumor cell lines [169].

### 9.1. Podoplanin and Cancer Stem Cells

Many tumors have a hierarchical organization where a subpopulation of self-renewing cancer stem cells (CSCs) or tumor-initiating cells (TICs) has the ability to differentiate into non-tumorigenic cells that constitute the bulk of the tumor [170]. CSCs are considered to be the cause of tumor recurrence, metastasis, and development of resistance to chemotherapy and radiotherapy [171,172]. Several cell-surface proteins, such as CD44, CD133, SOX2, and ALDH1, were proposed as markers of CSCs [172,173]. Atsumi and colleagues [174] first identified podoplanin as a novel marker of TICs in the human cervical SCC cell line A431. Isolated A431 podoplanin-positive cells could differentiate into podoplanin-negative cells, had higher colony formation efficiency, expressed increased CD44 levels, and were more tumorigenic than podoplanin-negative cells, fulfilling many properties of CSCs/TICs. It is well established that tumor cells grown in non-adherent conditions to form spheres are enriched in CSCs [175], and, although global podoplanin expression was reduced in A431 sphere-derived cells, fluorescence-activated cell sorting (FACS) analysis identified a subpopulation (about 5% cells) with high podoplanin expression that coincided with the side population (SP) characterized by efflux nuclear Hoechst fluorescent dye [176]. The SP phenotype is linked to the presence of proteins belonging to the ATP binding cassette (ABC) transporter family that contribute to multiple drug resistance in human cancer [172]. A431 podoplanin-positive cells exhibited lower cell death ratios than podoplanin-negative cells, accounting for its higher colony formation efficiency. This cell survival effect appeared to be mediated by podoplanin activation of the RhoA GTPase–ROCK signaling pathway through recruitment of ERM proteins [177].

In lung SCCs, the immunohistochemical staining of podoplanin and other stem-cell markers, such as CD44 and p63, revealed a hierarchical distribution pattern, with podoplanin mainly localized at the periphery of invading tumor nests co-expressed with CD44 and CD63, although these two proteins had wider distribution areas [157]. This hierarchical distribution pattern of primary tumors is also maintained in lymph node and pulmonary metastasis, which allowed the authors to propose that podoplanin-positive cells might be the TICs in the fraction of lung SCCs that express podoplanin, which corresponds to about 60% of tumors [178]. However, patients with primary tumors exhibiting this hierarchical distribution had better overall survival [157], and no differences on survival rates were observed between patients with and without this hierarchical distribution in lymph-node metastasis [178]. These facts are difficult to reconcile with podoplanin being a marker of CSCs in lung SCCs. Podoplanin was also proposed as a candidate marker for CSCs in esophageal SCCs [160,179] and gliomas/glioblastomas [180,181]. On the other hand, it was reported that co-expression of the CSC marker ALDH1 and podoplanin in pre-neoplastic oral leukoplakia is associated with a high risk of developing oral cancer [182].

### 9.2. Podoplanin in CAFs and Other Stromal Cells

There is evidence suggesting that CAFs, which are considered the most abundant stromal cells, represent a special type of “activated” fibroblast incorporated into the tumor microenvironment. CAFs strengthen malignant progression by releasing growth factors, cytokines, matrix proteins, and matrix-degrading proteases that stimulate angiogenesis and lymphangiogenesis and promote tumor cell proliferation, migration, and invasion [183,184]. Podoplanin was identified as a marker of CAFs in a variety of malignancies, including cancers in which it is absent from tumor cells, such as adenocarcinomas of breast, lung, or pancreas [9,185]. In most cancers, the prognostic value of podoplanin expression in CAFs for patient outcome is negative, associated with lymph node metastasis and shorter overall survival [9,154,185,186]. However, in tumors such as colorectal carcinomas and small-cell lung cancer (SCLC), podoplanin expression in CAFs is considered a favorable prognostic marker [187,188].

There are a number of functional studies that addressed the role of podoplanin-expressing CAFs on tumor development and progression (Table 4). Sorted podoplanin-positive CAFs enhanced the invasive potential of pancreatic carcinoma cell lines more effectively than podoplanin-negative CAFs, a result that is in accordance with clinical data showing that podoplanin expression in CAFs is a predictor of poor outcome in patients with invasive ductal pancreatic carcinomas [189]. Similarly, the presence of podoplanin-positive CAFs correlated with lymph node metastasis in patients with perihilar cholangiocarcinoma, and podoplanin expression in these CAFs was associated with increased migratory abilities [190]. Hoshino and colleagues [191] found that podoplanin-expressing CAFs, but not normal human fibroblasts enhanced the tumor formation ability of human lung adenocarcinoma cells, an effect mediated by podoplanin activation of RhoA–ROCK signaling in CAFs [192,193]. It seems that podoplanin-positive CAFs generate “tracks” in the extracellular matrix and promote cancer cell growth and invasion independently of MMP activity [193]. Again, these results are in agreement with clinical reports showing that podoplanin expression in CAFs is associated with a poor prognosis in patients with lung adenocarcinoma [194,195]. Other studies on lung adenocarcinomas showed that the presence of podoplanin-positive CAFs does not correlate with the mutation status of tumor cells [196], but is responsible for the resistance of lung adenocarcinoma cell lines to tyrosine kinase inhibitors of the epidermal growth factor receptor (EGFR), and for the lower overall response to EGFR inhibitors exhibited by patients with recurrent tumors harboring an EGFR-activating mutation with respect to patients with podoplanin-negative CAFs [197]. Interestingly, the podoplanin-mediated resistance of cancer cells to EGFR tyrosine kinase inhibitors depended on its cytoplasmic domain and required direct contact between CAFs and cancer cells [197], but the molecular mechanism for this effect remains to be elucidated. On the other hand, Luanpitpong and coworkers [198] showed that normal human lung fibroblasts can be activated to become podoplanin-expressing CAF-like cells by carbon nanotubes, a type of engineered nanomaterial used in the industrial field. These activated CAF-like cells promoted in vivo tumor growth and enhanced the number of CSCs in a non-SCLC cell line, and all these effects were podoplanin-dependent [198].

Podoplanin expression in CAFs is also a marker of poor prognosis for patients with breast cancer [199]. However, in vitro experiments failed to show a pro-tumorigenic effect of podoplanin-positive CAFs on breast cancer cells. Thus, forced podoplanin expression in normal human fibroblasts, although enhancing fibroblast motility, did not affect the migratory and invasive properties of breast cancer cells in co-culture experiments [200] (Table 4). An interesting observation in this report is that the numbers of podoplanin-positive CAFs were significantly higher in invasive ductal carcinomas with respect to non-invasive ductal carcinomas in situ, and these podoplanin-positive CAFs co-localized with blood vessels only in the former tumors. Overall, these results suggest that podoplanin expression in CAFs facilitates their migration into the stroma, where they may affect tumor vascularization [200]. The presence of podoplanin-positive CAFs was also found in synchronous lymph-node metastasis produced by HER2-positive metastatic breast carcinomas. These cells represent a population of CAFs that migrated from the primary tumor rather than resident fibroblast-like stromal cells [201]. In addition, Tejchman and colleagues [27] proposed that, in a hypoxic environment, podoplanin-positive CAFs facilitate the escape of CCR7-positive breast cancer cells from immunosurveillance by binding of podoplanin to CCL21. Sequestering of CCL21 by podoplanin would impair adhesion of CCR7-expressing natural killer (NK) cells to endothelial cells. Nevertheless, this hypothesis is supported only by in vitro experiments. In line with these results, Cremasco and coworkers [202] found that immune cells, particularly CD8^+^ cytotoxic T lymphocytes, infiltrating the periphery of mouse breast tumors, were in close contact with podoplanin-expressing CAFs. These podoplanin-positive fibroblasts, which express a genetic signature resembling FRCs, generated a dense ECM, preventing immune cells from going deeper in the tumor parenchyma, and inhibiting T-cell proliferation in a nitric oxide-dependent manner [202].

In contrast to the above results, podoplanin-expressing CAFs inhibited the proliferation of SCLC cells in co-culture experiments, and, when tumor cells were co-injected with either podoplanin-positive or podoplanin-negative CAFs in mice, differences in the growth of tumors were statistically non-significant [203]. This result is in concordance with previous studies suggesting that presence of podoplanin-expressing CAFs in SCLC has a favorable prognostic value [188] (Table 4). A rational explanation for the different behavior of podoplanin-expressing CAFs as promoters or inhibitors of malignancy is still lacking.

Ectopic lymphoid-like structures formed by lymph-node stromal cells not only play an important role in the regulation of the inflammatory response (see Section 8), but also contribute to tumor development and progression. In this context, a recent report found that podoplanin-expressing lymph-node stromal cells promote melanoma growth in vivo by inhibiting anti-tumor specific CD4^+^ T-cell proliferation and inducing the death of tumor infiltrating CD4^+^ lymphocytes in a cell-contact-independent fashion [204]. This observation points to a novel role of podoplanin-expressing lymph node stromal cells in tumor immune evasion. Nevertheless, the mechanism for recruitment of podoplanin-expressing lymph node stromal cells in the tumor microenvironment is not known, and the nature of the cell(s) responsible for these effects is also an unsolved question, since podoplanin-expressing lymph node stromal cells are likely composed of a heterogeneous mixture of FRCs, LECs, and Th17 cells.

## 10. Podoplanin as a Promoter of Malignancy

**Figure 3:**
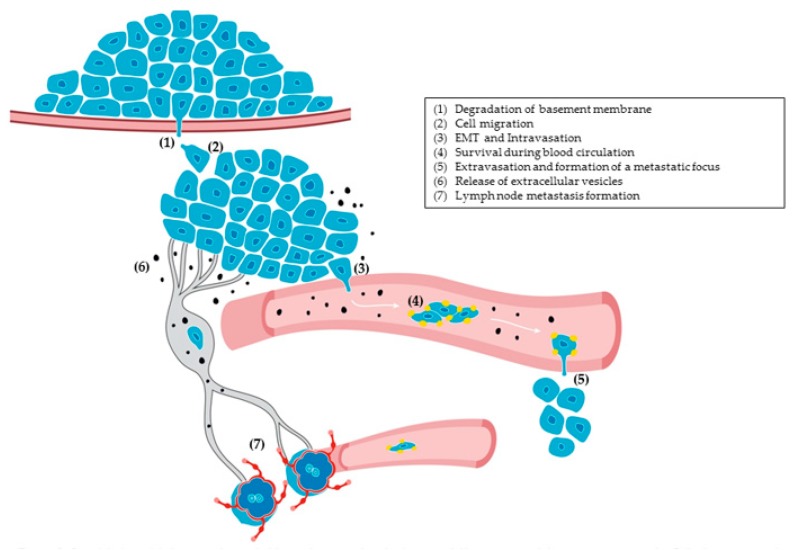
Simplified model showing the probable implication of podoplanin in different steps of the metastatic cascade. Cells from a premalignant tumor traverse the basement membrane using the proteolytic activity of invadopodia (1) and invade the connective issue (2) to form a malignant tumor. Tumor cells can undergo epithelial–mesenchymal transition (EMT) and enter into the blood stream by degrading the basement membrane of the blood vessel through invadopodia (3). Malignant cells resist against shear stress and inhibit immune cell assaults by activating and aggregating platelets (4). Platelet aggregation favours tumor-cell adhesion and emboli formation in the microvasculature; adhered tumor cells use invadopodia to extravasate from the blood vessel (5). Malignant cells secrete soluble factors and extracellular vesicles (6) that promote angiogenesis and lymphangiogenesis, and infiltrate pre-existing lymphatic vessels to reach the lymph nodes and form metastasis (7).

Significant insights into the role of podoplanin as a promoter of malignancy came from functional studies carried out by different laboratories. Overall, the main conclusion from these experiments was that podoplanin promotes tumor progression through a variety of strategies encompassing different steps of the metastatic cascade [154,205] (Figure 3).

### 10.1. Podoplanin Promotes Tumor-Cell Migration and EMT

In cultured cells, podoplanin is concentrated at cell-surface protrusions, i.e., microvilli, filopodia, and ruffles [13,14,35]. These dynamic plasma membrane structures are shaped by the actin cytoskeleton and are involved in cell motility and migration. Podoplanin was found to induce filopodial extensions and cell motility by recruiting ezrin and promoting a rearrangement of the actin cytoskeleton [13,14,206]. Podoplanin is often expressed at the invasive front of SCCs where it usually co-localizes with the cell–cell adhesion molecule E-cadherin [206]. Nevertheless, podoplanin expression was found sometimes to correlate with downregulation of E-cadherin expression in some areas of oral SCCs [14,207]. A similar correlation was found in peritumoral keratinocytes of extramammary Paget’s disease, a rare and life-threatening skin carcinoma arising from the mammary gland [163]. These results suggest that podoplanin expression in tumors in vivo may be linked to weakened cell–cell contacts. The ability of podoplanin to disrupt cell–cell adhesion was clearly demonstrated in vitro, as ectopic expression of podoplanin in human immortalized HaCaT keratinocytes allowed delocalization of E-cadherin out of cell–cell contacts and reduced calcium-dependent intercellular adhesiveness [14]. Moreover, forced expression of podoplanin in mouse premalignant MCA3D keratinocytes and Madin-Darby canine kidney (MDCK) epithelial cells induced a full EMT with loss of epithelial markers, such as E-cadherin and keratins, and upregulation of mesenchymal proteins, such as N-cadherin and vimentin [13,35].

EMT in the invasive front of carcinomas is associated with delamination of cancer cells from the primary tumor, i.e., the first step on the metastatic cascade, allowing tumor cells to colonize other tissues [208] (Figure 3). In fact, podoplanin-expressing cells that elicited an EMT exhibited increased migratory and invasive capacities in vitro and produced metastasis in lymph nodes in vivo [13,96]. An intriguing observation from these studies was the transforming capacity exhibited by podoplanin in epithelial cells. Podoplanin expression in non-tumorigenic cultured mouse keratinocytes conferred on these cells the ability to produce tumors upon injection in nude mice [96]. The oncogenic properties of podoplanin were confirmed in human oral SCC cell lines expressing endogenous podoplanin, as its knockdown significantly reduced the tumorigenic capacities of HN2 and HN5 oral carcinoma cells [169,209]. An explanation for the oncogenic behavior of podoplanin came from proteomic studies in which upregulation of pro-oncogenic proteins and downregulation of tumor suppressors was observed in MDCK cells overexpressing human podoplanin [169]. On the other hand, results from different laboratories suggest that cancer cells eliciting EMT acquire characteristics of CSCs [210]. However, it is unclear whether podoplanin-induced EMT is associated with the generation of stem-like characteristics. In this respect, Bresson and colleagues [61] found that podoplanin is highly expressed in triple-negative breast carcinomas induced in transgenic mice expressing constitutively active β-catenin in mammary basal cells. In this context, deletion of podoplanin attenuated tumorigenesis and formation of tumor-spheres in culture, which is compatible with TIC depletion. Interestingly, tumors developed in the absence of podoplanin were more differentiated and showed signs of a mesenchymal–epithelial transition, the reverse process of EMT [61].

Podoplanin-induced EMT requires its binding to ERM proteins through a juxtamembrane cluster of basic amino acids within the cytosolic domain, leading to the activation of RhoA GTPase–ROCK signaling [35]. Another requisite for podoplanin to promote EMT and cell migration is its association to membrane DRM fractions or lipid rafts. The localization of podoplanin in lipid rafts is mediated by a motif (G^133^IIVG^137^ in human podoplanin) in the transmembrane region, corresponding to the consensus sequence GXXXG, which also mediates podoplanin self-assembly [28]. This motif was highly conserved during evolution and was subjected to positive selection, pointing to its functional relevance [8]. Either mutation or substitution of this region allowed exclusion of the glycoprotein from lipid rafts and blocked podoplanin-induced EMT [28]. As mentioned above (Section 2.2), podoplanin interacts with CD44 at the leading edge of SCC cells, but not in cell–cell contacts [33]. CD44 also binds ERM proteins [34], but its interaction with podoplanin appears to be ERM-independent. Gain- and loss-of-function experiments suggest that podoplanin cooperates with CD44 to promote directional migration of carcinoma cells [33].

Podoplanin was also found to promote collective cell migration and invasion in vitro and in vivo. Thus, Wicky and coworkers [206] reported that podoplanin stimulates MCF7 breast cancer cell migration in the absence of EMT, an event linked to downregulation of RhoA, RhoC, and Cdc42 GTPase activities. In addition, these authors used a transgenic mouse model of pancreatic cancer to show that podoplanin promotes tumor invasion without affecting tight junctions and E-cadherin-mediated cell–cell contacts [206]. Downregulation of RhoA and upregulation of Cdc42 GTPase activities was also associated with podoplanin-mediated stimulation of cell motility and invasiveness in oral SCC cells [211], although it is not clear in this report whether podoplanin-mediated invasion is collective or involves a partial EMT. Overall, these reports suggest that regulation of Rho GTPase signaling pathways by podoplanin is cell-context-specific. Podoplanin-mediated stimulation of collective cell migration agrees with the fact that, more often, podoplanin co-localizes with E-cadherin at the invasive front of human carcinomas [14,206]. Nevertheless, it should be taken into account that physiological and pathological EMT is not an all-or-nothing process, but intermediate EMT states exist that are associated with loosening of cell–cell adhesiveness and not with the loss of a particular epithelial marker [208].

### 10.2. Podoplanin Promotes ECM Remodeling and Cancer Cell Invasion

Podoplanin expression was found to correlate, and even co-localize, with MMPs in invading tumors, an event associated with an aggressive behavior [211,212,213]. Furthermore, podoplanin was reported to upregulate membrane-bound MMP14 (also known as MT1-MMP), associated with stimulation of oral SCC cell invasion, via a mechanism involving activation of the small GTPase Cdc42 [211]. Interestingly, podoplanin and MMP14 appear to form a complex at the leading edge of migrating cells, since they co-localized at cell-surface protrusions and co-precipitated together in immunoprecipitation experiments [211]. Cancer cell invasion not only depends on the expression/secretion of MMPs by tumor cells, since stromal cells (particularly CAFs) were also found to contribute to cancer metastasis through MMPs [184]. Thus, Li and coworkers [214] reported that podoplanin stimulates TGF-β secretion in oral SCC cells, which activates surrounding fibroblasts upregulating MMP2 and MMP14 expression. Activated CAFs, in turn, induce podoplanin expression in carcinoma cells through the TGF-β–Smad pathway (Table 1 and Table 2) and enhance cell invasion by activating EGFR, AKT, and ERK signaling. The TGF-β–Smad pathway was also found to stimulate podoplanin expression in fibrosarcoma cells [215].

In order to invade and metastasize, cancer cells must degrade and remodel the ECM, including both the basement membrane and the interstitial matrix. The basement membrane is a dense, sheet-like specialized type of ECM underlying epithelia and endothelial cells, which provides a barrier separation from neighbor tissues, a substrate for cell adhesion, and a platform for biochemical and mechanical signaling between the epithelium/endothelium and external environment [216]. Cancer cells from primary tumors form actin-rich membrane protrusions with focalized proteolytic activity, called invadopodia, which degrade the ECM and perforate basement membranes allowing local invasion and, eventually, entry into blood vessels [217] (Figure 3). Similar structures called podosomes are found in a variety of normal cell types, such as osteoclasts, macrophages, and endothelial cells. Because invadopodia and podosomes share most of their structural components, have a similar architecture, and are functionally equivalent, they were unified under the name of invadosomes [218]. Typical components of invadopodia and podosomes are actin dynamics regulators, such as cortactin, cofilin, and members of the Wiskott–Aldrich syndrome protein (WASP) family, scaffolding proteins as Tks5, integrins, and MMPs, such as MMP14, MMP2 and MMP9.

We and others reported that podoplanin is a novel component of invadopodia in breast cancer and SCC cells [38,219]. Podoplanin is absent from podosomes, but is located in the adhesion ring of invadopodia, which is formed by the core of polymerized actin (actin puncta) and integrins. Recruitment of podoplanin to the adhesion ring of invadopodia requires binding to ERM proteins and association with lipid rafts [38]. Functional studies suggest that podoplanin is not involved in the formation of invadopodia, but promotes their maturation and stabilization by activating the RhoC–ROCK–LIM kinase–cofilin signaling pathway, facilitating an efficient degradation of the ECM [38]. Interestingly, CD44 was also identified as a component of invadopodia in breast cancer cells, where it appears to be involved in their formation and recruitment of MMP14 [220,221]. In addition, CD44 was implicated in the formation of invadopodia-like protrusions in glioblastoma CSCs by mediating ECM sensing [222]. Since glioblastoma CSCs express podoplanin [180,181], CD44 is present in SCCs [33], and podoplanin binds both CD44 [33] and MMP14 [211] at the surface of cancer cells, it should be of great interest to explore the role of the podoplanin–CD44 interaction in invadopodia assembly and maturation, and the recruitment of MMP14 to these membrane structures. On the other hand, there is evidence suggesting that exosomes are critical for invadopodia formation and degradative activity, which in turn are key sites for exosome release [223]. In this respect, we found that podoplanin knockdown in oral SCC cells diminished both invadopodia-mediated proteolysis [38] and exosome secretion [169], suggesting a role for podoplanin as a mediator of the synergistic association between invadopodia and exosomes in these cells.

### 10.3. Does Podoplanin Promote Tumor Lymphangiogenesis and Angiogenesis?

The first indicator of tumor progression in most epithelial malignancies is lymph node metastasis. Many tumors activate lymphangiogenesis within the tumor, which results in increased density of peritumoral and/or intratumoral lymphatic vessels [7,224]. The presence of lymphangiogenesis in tumor-draining lymph nodes is also observed even before cancer cells arrive to regional or sentinel lymph nodes. Lymph-node lymphangiogenesis is believed to facilitate cancer dissemination to distant tissues and acts as a permissive lymphovascular niche for the survival of metastatic cells [7].

There are contradictory results with respect to the involvement of podoplanin in tumor lymphangiogenesis. Cueni and co-workers [225], using a human breast carcinoma xenograft model, found that podoplanin expression enhanced lymphangiogenesis and metastasis to regional lymph nodes without affecting primary tumor growth. We also showed that ectopic expression of podoplanin in pre-malignant mouse keratinocytes promoted lymph-node metastasis upon injection into mice [96]; however, whether or not this was associated with enhanced lymphangiogenesis in the primary tumor or in the lymph nodes was not analyzed. In addition, several reports linked podoplanin expression in CAFs of prostate, bladder, ovary, pancreas, and breast carcinomas with intratumoral lymphangiogenesis, lymph node metastasis, and lymphatic invasion [185,186]. In most of these reports, the mechanism via which podoplanin stimulates lymphangiogenesis was not addressed, but it may involve podoplanin-mediated secretion of pro-lymphangiogenic growth factors or cytokines, such as VEGF-C, VEGF-D, and CCL21. In particular, CCL21, which binds podoplanin with high affinity and is secreted by LECs and tumor cells, could facilitate the access of tumor cells to lymphatic vessels [7,10]. In contrast, Suzuki and colleagues [226] showed that podoplanin expression in a lung squamoid cancer cell line restrained lymph node metastasis and suppressed lymphangiogenesis, in association with downregulation of VEGF-C.

Tumor cells and CAFs also secrete extracellular vesicles (microparticles, microvesicles, exosomes), containing proteins and RNA, into the tumor microenvironment, which promote malignancy via different mechanisms [227,228]. In contrast to microvesicles or microparticles that are formed via direct budding of the plasma membrane, exosomes originate from the endocytic pathway via inward budding of the limiting membrane of late endosomes (multivesicular bodies) to form intraluminal vesicles, which are released to the extracellular environment upon fusion of the multivesicular bodies with the plasma membrane. We reported that podoplanin is a component of exosomes and microvesicles, and that podoplanin-containing exosomes stimulated both angiogenesis and lymphangiogenesis in vitro, but only the latter was dependent on podoplanin present at the surface of vesicles [169]. The question whether exosomal podoplanin stimulates lymphangiogenesis in vivo remains to be investigated.

There is also evidence indicating a role for podoplanin in tumor angiogenesis. Thus, podoplanin was able to induce the expression of fibroblast growth factor (FGF)-1, a pro-angiogenic factor, in a human lung SCC cell line, and co-expression of podoplanin and FGF-1 correlated with increased vascularization of tumors and shorter overall survival of lung SCC patients [229]. Likewise, the conditioned medium obtained from glioma cells overexpressing podoplanin strongly induced angiogenesis in vitro with respect to mock transfected cells. However, this effect was not associated with increased production of angiogenic factors, such as VEGF, VEGF-C, and VEGF-D [230]. Nonetheless, whether or not this pro-angiogenic effect resides in the particulate (extracellular vesicles) or soluble fraction of the conditioned medium is unknown.

### 10.4. Podoplanin–CLEC-2 Interaction in Hematogenous Metastasis and Cancer-Associated Thrombosis

CLEC-2 is highly expressed in megakaryocytes and platelets, and podoplanin expressed on the surface of tumor cells induces platelet activation by binding to CLEC-2 (see above Section 2.1 and Section 3.1). Platelets favor tumor dissemination and metastasis by protecting tumor cells from mechanical stress during bloodstream circulation, enabling them to evade from immune surveillance, and mediating adherence to the vascular endothelium, favoring embolization of the microvasculature and extravasation. In addition, activated platelets release bioactive factors, such as TGF-β and PDGF, which facilitate extravasation of circulating tumor cells and promote the growth of metastatic foci [231,232,233]. Experimental evidence demonstrating the importance of the podoplanin–CLEC-2 interaction for tumor spread is ample and strong. For example, neutralizing Abs targeting either podoplanin or CLEC-2 are able to block platelet aggregation and metastasis induced by podoplanin-expressing tumor cells [15]. Also, pulmonary metastases of podoplanin-positive, but not of podoplanin-negative tumors were greatly inhibited in CLEC-2-deficient mice [234]. Finally, CD9 tetraspanin prevented the podoplanin–CLEC-2 interaction and inhibited platelet aggregation and metastasis of fibrosarcoma cells [31]. Interestingly, CD9 is considered as a suppressor of metastasis in a variety of cancers, including breast, non-SCLC, colon, and myeloma, although it was shown to promote malignancy in other cancer types [235].

Because of its ability to induce platelet aggregation, podoplanin was identified as a risk factor for coagulation and thrombosis in inflammatory processes (see Section 5) and cancer, mostly in patients with brain tumors and leukemias [236,237,238]. Venous thromboembolism is a frequent life-threatening complication in cancer patients [239,240]. For example, about 14% of patients with primary brain tumors (mostly high-grade gliomas) develop venous thromboembolism, an event strongly associated with overexpression of podoplanin [236]. Lavallée and colleagues [238], using an in vivo xenograft model, found that promyelocytic leukemia cells expressing podoplanin induced thrombocytopenia and prolonged bleeding time, while none of these effects were observed with podoplanin-negative leukemia cells. Podoplanin was also found to mediate platelet aggregation and thrombocytopenia induced by umbilical cord mesenchymal stem cells infused into mice via the tail vein. In contrast, bone-marrow-derived mesenchymal stem cells, which do not express podoplanin, were unable to activate platelets or induce changes in platelet count [241]. Since mesenchymal stem cells are thought to be ideal vehicles for delivery of therapeutic tools for cancer treatment [242], this study identified that some mesenchymal stem cells with particular adhesive properties may be thrombotic.

With respect to brain tumors, the following question emerges: how can podoplanin expressed on the surface of tumor cells reach the bloodstream and trigger venous thromboembolism? There are a number of potential mechanisms for this [240]. Firstly, a soluble form of podoplanin can be shed from the cell surface into the blood circulation. In this regard, Cueni and coworkers reported that targeted expression of the podoplanin ectodomain in the epidermis of transgenic mice allowed entrance of the protein into the blood circulation and induced microthrombi and thrombocytopenia, with occasional fatal hemorrhages [121]. Secondly, podoplanin can be released from tumor cells attached to the membrane of extracellular vesicles [168,169], and circulating microparticles are associated with venous thromboembolism in cancer patients [239]. Thirdly, circulating tumor cells (CTCs) are frequently detected in the blood of glioblastoma patients [243] and, thus, podoplanin-expressing CTCs can detach from the primary tumor and enter the bloodstream. In line with this hypothesis, podoplanin-positive CTCs were identified in the blood circulation associated with poor prognosis of head and neck SCC patients [244]. Altogether, these studies point to podoplanin as a potential biomarker to predict and identify patients at increased risk of cancer-associated venous thromboembolism [240].

All these findings enlighten the podoplanin–CLEC-2 axis as a novel therapeutic target for treatment of cancer and its associated thromboembolism. To this aim, an array of monoclonal antibodies (mAbs) directed against the extracellular domain of podoplanin was developed in order to disrupt the podoplanin–CLEC-2 interaction and inhibit platelet aggregation and hematogenous metastasis in vivo [15,37,240]. Particularly interesting for therapeutic purposes is the LpMab series recognizing cancer-specific aberrant glycosylated podoplanin, which reacts only with podoplanin expressed in tumors and not with podoplanin in normal cells [245,246,247]. A novel strategy to neutralize podoplanin function in metastasis is based on chimeric antigen receptor (CAR) T-cell immunotherapy [248]. Shiina and colleagues [249] used peripheral blood monocytes transduced with a CAR containing a podoplanin-specific mAb to generate CAR T cells against podoplanin-expressing glioblastomas. Systemic infusion of these CAR T cells into immunodeficient mice inhibited the growth of intracranial glioma xenografts in vivo, pointing to podoplanin as a potential target to treat glioblastomas by adoptive immunotherapy. In addition, platelet antagonists targeting CLEC-2, such as the derivative of 4-*O*-benzoyl-3 methoxy-betanitrostyrene 2CP [250] and cobalt hematoporphyrin [251] were developed and found to inhibit the podoplanin–CLEC-2 interaction, as well as metastasis and thrombosis.

## 11. Future Directions

While the research about the implication of podoplanin in patho-physiological processes, such as immune response, thrombosis, inflammation, and cancer, experienced notable advances in the last decade, there are still many issues for which we have incomplete knowledge. Many unanswered questions regarding the biochemistry of podoplanin persist. For example, are there additional physiological partners to be discovered for this molecule? Is it phosphorylated in vivo and, if so, under which circumstances? Does the proteolytic processing of podoplanin have functional relevance? Is it regulated by phosphorylation? Podoplanin was defined by several laboratories as a marker of CSCs. However, its functions with regards to the maintenance and/or biological activity of CSCs, if any, are far from being understood. Similarly, it is well established that the upregulation of podoplanin expression in subsets of activated stromal cells appears to be a general response during inflammation and cancer; however, the contribution of podoplanin to the pathogenicity of many inflammatory diseases and types of cancer is controversial. In this respect, much more effort should be put in clarifying the function of podoplanin with regards to effector T cells, myofibroblast-like cells, and CAFs, as well as in elucidating the involvement of podoplanin in lymphangiogenesis. Following these and other avenues will significantly improve our knowledge on the function of podoplanin in inflammation and cancer in the near future.

## Figures and Tables

**Table 1:** Cytokines and other compounds stimulating podoplanin expression.

Cytokine/Compound	Cell Type	Reference
LTβR ligands	Reticular cell (FRC, FDC)	[78]
TNF-α	Macrophage Monocyte Synovial fibroblast Dermal fibroblast	[85] [111] [139,141] [149]
TGF-β1	Keratinocyte Synovial fibroblast SCC Fibrosarcoma	[89] [139] [214] [215]
IFN-α	Keratinocyte	[89]
VEGF-C	Monocyte	[111]
IL-1β	Synovial fibroblast Dermal fibroblast	[139,141] [149]
IL-3	LEC BEC ^1^ Monocyte	[109] [110] [111]
IL-6	Keratinocyte	[89]
IL-7	EC ^2^	[108]
IL-22	Keratinocyte	[89]
LPS	Macrophage Monocyte	[85] [111]
Fibronectin	Monocyte	[111]
TPA	Keratinocyte Dermal fibroblast	[88,93] [88]

^1^ At a low level; ^2^ Endothelial cells with mixed vascular and lymphatic characteristics. LPS: lipopolysaccharide; TPA: 12-*O*-tetradecanoylphorbol 13-acetate; FRC: fibroblast-like reticular cells; FDC: follicular dendritic cell; SCC: squamous cell carcinoma; LEC: lymphatic endothelial cell; BEC: blood endothelial cell.

**Table 2:** Signaling pathways found to control podoplanin expression in different cell types.

Pathway	Regulation	Cell Type	Reference
JAK/STAT	Up	Keratinocyte	[89]
TGF-β/Smad	Up Up Up	Keratinocyte SCC Fibrosarcoma	[89] [214] [215]
NF-κB	Up	Keratinocyte	[94]
Notch	Down	LEC	[107]

LEC: Lymphatic endothelial cell; SCC: squamous cell carcinoma.

**Table 3:** Involvement of podoplanin (PDPN) in autoimmune diseases.

Disease	Cell Types Found to Have Upregulated PDPN Expression	Role in Pathogenicity
Multiple sclerosis	Th17	Promotes (mouse)? Protects (human)?
Rheumatoid arthritis	Th17 Synoviocytes	Promotes Promotes
Systemic sclerosis	Fibroblasts	?
Sjögren syndrome	MEC Lymphocytic infiltrate	? Promotes?
Discoid lupus erythematosus of the lip	Oral mucosa	Promotes progression to SCC

MEC: Myoepithelial cell; SCC: squamous cell carcinoma; ?: unclear or not investigated.

**Table 4:** Role of podoplanin-expressing cancer-associated fibroblasts (CAFs) in tumor progression. Correlation between functional studies and clinical data.

Cancer Type	Functional Studies	Clinical Significance (Prognosis)	References
Pancreatic ductal carcinoma	Promotes tumor cell invasiveness	Poor	[189]
Lung adenocarcinoma	Promotes tumorigenicity and chemoresistance	Poor	[191,192,193,194,195,197,198]
SCLC	Inhibits tumor cell growth	Favorable	[188,203]
Breast carcinoma	No effect on tumor cell invasiveness; Promotes immunosppression	Poor	[27,199,200,201,202]
Perihilar cholangiocarcinoma	Promotes tumor cell migration	Poor	[190]

SCLC: Small cell lung carcinoma.
